# TPLATE Recruitment Reveals Endocytic Dynamics at Sites of Symbiotic Interface Assembly in Arbuscular Mycorrhizal Interactions

**DOI:** 10.3389/fpls.2019.01628

**Published:** 2019-12-20

**Authors:** Giulia Russo, Gennaro Carotenuto, Valentina Fiorilli, Veronica Volpe, Antonella Faccio, Paola Bonfante, Mireille Chabaud, Marco Chiapello, Daniel Van Damme, Andrea Genre

**Affiliations:** ^1^Department of Agricultural, Forest and Food Sciences, University of Torino, Torino, Italy; ^2^Department of Life Sciences and Systems Biology, University of Torino, Torino, Italy; ^3^Institute for Sustainable Plant Protection, National Research Council, Torino, Italy; ^4^LIPM, Université de Toulouse, INRAE, CNRS, Castanet-Tolosan, France; ^5^Department of Plant Biotechnology and Bioinformatics, Ghent University, Ghent, Belgium; ^6^VIB Department of Plant Systems Biology, Ghent University, Ghent, Belgium

**Keywords:** arbuscular mycorrhizas, *Medicago truncatula*, *Daucus carota*, endocytosis, symbiosis, live cell imaging, confocal laser scanning microscope, transmission electron microscope

## Abstract

**Introduction:** Arbuscular mycorrhizal (AM) symbiosis between soil fungi and the majority of plants is based on a mutualistic exchange of organic and inorganic nutrients. This takes place inside root cortical cells that harbor an arbuscule: a highly branched intracellular fungal hypha enveloped by an extension of the host cell membrane—the perifungal membrane—which outlines a specialized symbiotic interface compartment. The perifungal membrane develops around each intracellular hypha as the symbiotic fungus proceeds across the root tissues; its biogenesis is the result of an extensive exocytic process and shows a few similarities with cell plate insertion which occurs at the end of somatic cytokinesis.

**Materials and Methods:** We here analyzed the subcellular localization of a GFP fusion with TPLATE, a subunit of the endocytic TPLATE complex (TPC), a central actor in plant clathrin-mediated endocytosis with a role in cell plate anchoring with the parental plasma membrane.

**Results:** Our observations demonstrate that *Daucus carota* and *Medicago truncatula* root organ cultures expressing a 35S::*At*TPLATE-GFP construct accumulate strong fluorescent green signal at sites of symbiotic interface construction, along recently formed perifungal membranes and at sites of cell-to-cell hyphal passage between adjacent cortical cells, where the perifungal membrane fuses with the plasmalemma.

**Discussion:** Our results strongly suggest that TPC-mediated endocytic processes are active during perifungal membrane interface biogenesis—alongside exocytic transport. This novel conclusion, which might be correlated to the accumulation of late endosomes in the vicinity of the developing interface, hints at the involvement of TPC-dependent membrane remodeling during the intracellular accommodation of AM fungi.

## Introduction

Arbuscular mycorrhizal (AM) symbiosis with Glomeromycotina fungi supports life of most land plants, including the majority of crop species, providing their roots with a more efficient access to soil nutrients (Gutjahr and Parniske, 2013). In change, host plants share with their symbiotic fungi up to 20% of photosynthesis-derived carbon, in form of sugars and lipids (Bago et al., 2002; McLean et al., 2017). This nutrient exchange takes place in highly branched hyphae, called arbuscules, that are accommodated inside the living root cells (Gutjahr and Parniske, 2013). This intimate intracellular interaction is achieved through the assembly of a novel host cell compartment, called the symbiotic interface (Gutjahr and Parniske, 2013).

Enveloped by the perifungal membrane, a specialized extension of the host plasmalemma (Pumplin and Harrison, 2009), the symbiotic interface surrounds intracellular hyphae and arbuscules with plant cell wall-related compounds.

Furthermore, live cell investigations have revealed how the symbiotic interface is assembled within the prepenetration apparatus (PPA), a broad cytoplasmic aggregation (Genre et al., 2005; Genre et al., 2008), where the secretory process is focused and coordinated (Genre et al., 2012).

We have recently demonstrated that root colonization by AM fungi is associated with cell cycle reactivation (Carotenuto et al., 2019a; Carotenuto et al., 2019b), suggesting that symbiotic interface biogenesis could evolutionarily and developmentally be related to cell plate assembly (Russo et al., 2019).

Indeed, analogous cellular features have been described for the two processes. Firstly, the composition of the interface materials has been described as largely analogous to that of the cell plate, the non-structured cell wall that divides daughter cells at the end of mitosis (Balestrini and Bonfante, 2005; Balestrini and Bonfante, 2014). Secondly, ultrastructural details of the PPA aggregate present remarkable similarities with the organization of subcellular compartments during cell plate assembly (Genre et al., 2012). This includes the concentration of Golgi stacks and proliferation of trans-Golgi membranes (Genre et al., 2012). In this frame, the occurrence of late endosomes/multivesicular bodies (MVB) in the PPA aggregate has raised the question whether the PPA-driven exocytic activity could be associated with endocytic processes (Genre et al., 2008; Genre et al., 2012). Indeed, the massive exocytic process directed to the cell plate by phragmoplast microtubules during plant cell division (Lee and Liu, 2013; Boruc and Van Damme, 2015) is associated with extensive endocytic recycling of surplus membrane (Backues et al., 2007). In fact, a key actor of the endocytic process—the adaptin-related protein TPLATE, a subunit of the octameric TPLATE adaptor complex (TPC), has been shown to accumulate on both the cell plate membrane and the plasmalemma at the cortical division zone surrounding the cell plate insertion site, where the cell plate will eventually fuse (Van Damme et al., 2006; Van Damme et al., 2011; Gadeyne et al., 2014). *TPLATE* and other endocytic players (Gadeyne et al., 2014) have been shown to be upregulated during early AM development (Russo et al., 2019), and TPLATE-GFP-decorated cell walls have highlighted the occurrence of ectopic cell divisions in the root area that is preparing to accommodate arbuscules (Russo et al., 2019).

The present study, largely based on live cell imaging of *D. carota* and *M. truncatula* ROCs colonized by the AM fungus *Gigaspora gigantea*, suggests PPA-associated endocytic activity at the sites of perifungal membrane assembly. In particular, the endocytic marker accumulates at the growing tips of the perifungal membrane and at sites of perifungal membrane fusion with the peripheral plasmalemma, in striking analogy with the described TPLATE localization during cell plate expansion and fusion. Our results support the involvement of intense endocytic processes, likely related to perifungal membrane modeling and the recycling of membrane surplus at sites of hyphal exit from the host cell.

## Methods

### Plant and Fungal Materials

*Agrobacterium rhizogenes*-transformed root organ cultures (ROCs) expressing the *35S::AtTPLATE-GFP* vector (Van Damme et al., 2004) were obtained from *Medicago truncatula* Jemalong A17 wild-type and *dmi3-1* seedlings (Sagan et al., 1995; kindly provided by M. Chabaud, LIPM, INRA, Toulouse, France), according to Boisson-Dernier et al. (2001). ROCs from *Daucus carota* var Sativus expressing the same vector were obtained according to Bécard and Fortin (1988). For both species, transformed roots with a high level of fluorescence were selected 21 days after transformation, decontaminated and subcultured on M medium (Bécard and Fortin, 1988) at 25°C in the dark for subsequent use as ROCs. ROC generation was repeated in two independent experiments for each species and line, with overlapping results in terms of GFP fluorescence pattern. In each case, a single representative clone was chosen for further studies. Transformation efficiency and expression of *35S::AtTPLATE-GFP* was checked with GFP specific primers on both genomic DNA and cDNA obtained from all the selected lines, as described in Russo et al. (2019).

*G. gigantea* (isolate HC/FE30, Herbarium Cryptogamicum Fungi, University of Torino, Italy), which is characterized by strong cytoplasmic autofluorescence (Genre et al., 2005), was used to inoculate ROCs *in vitro* for confocal imaging. Spores of *G. gigantea* were collected from pot cultures in sand (with leek and clover, respectively), surface-sterilized, and stored at 4°C according to Bécard and Fortin (1988) until inoculation.

### Confocal Microscopy

The targeted AM inoculation technique for studying early stages of the symbiotic association between *Gigaspora* species and transformed root cultures, developed by Chabaud et al. (2002) and adapted for confocal observation by Genre et al. (2005), was applied to both *M. truncatula* and *D. carota* ROCs expressing *35S::AtTPLATE-GFP*. An upright Leica TCS SP2 confocal microscope fitted with a long distance 40X water-immersion objective (HCX Apo 0.80) was used for imaging living ROCs directly in the Petri dishes. The argon laser band of 488 nm was used to excite both GFP and *G. gigantea* autofluorescence. The two signals were separated using specific emission windows: 500 to 525 nm for GFP and 590 to 630 nm for fungal autofluorescence. The latter channel was then false-colored in red to maximize the contrast in overlapping images.

Confocal images presented in figures are representative of the observations performed on a minimum of 30 infection units from at least 10 independent ROC specimens for each plant species.

### Electron Microscopy

Following the identification of GFP-labeled PPAs in the inner cortex through confocal microscopy, five *D. carota* ROC segments were excised and processed for transmission electron microscopy (TEM) according to Genre et al. (2008). After fixation, samples were infiltrated and embedded in a thin layer of Epon-Araldite (Hoch, 1986) resin. Fungal colonization sites within flat-embedded samples were selected under an optical microscope, excised with a razor blade, and mounted on resin stubs prior to ultramicrotomy. Ultrathin (70 nm) sections were cut, counterstained, and observed using a Philips CM10 TEM.

## Results

### TPLATE-GFP Accumulates in Epidermal PPA

TPLATE localization was studied through the expression of a 35S::*At*TPLATE-GFP fusion protein in *M. truncatula* and *D. carota* ROCs. This construct had previously been used in both species to mark cell divisions in meristematic and differentiated root tissues (Russo et al., 2019). The same GFP fusion was also expressed in ROCs derived from the non-mycorrhizal *dmi3-1* mutant of *M. truncatula* (Sagan et al., 1995; Levy et al., 2004), where the loss of *Does not Make Infection 3* (a nuclear localized calcium-and-calmodulin-dependent kinase) blocks a symbiotic signal transduction pathway and halts fungal colonization to the surface of epidermal cells (Morandi et al., 2005). ROCs were colonized with the autofluorescent AM fungus *G. gigantea* and dozens of infection sites, at different stages of root colonization, were observed for each experimental condition through confocal live cell imaging.

**Figure 1:**
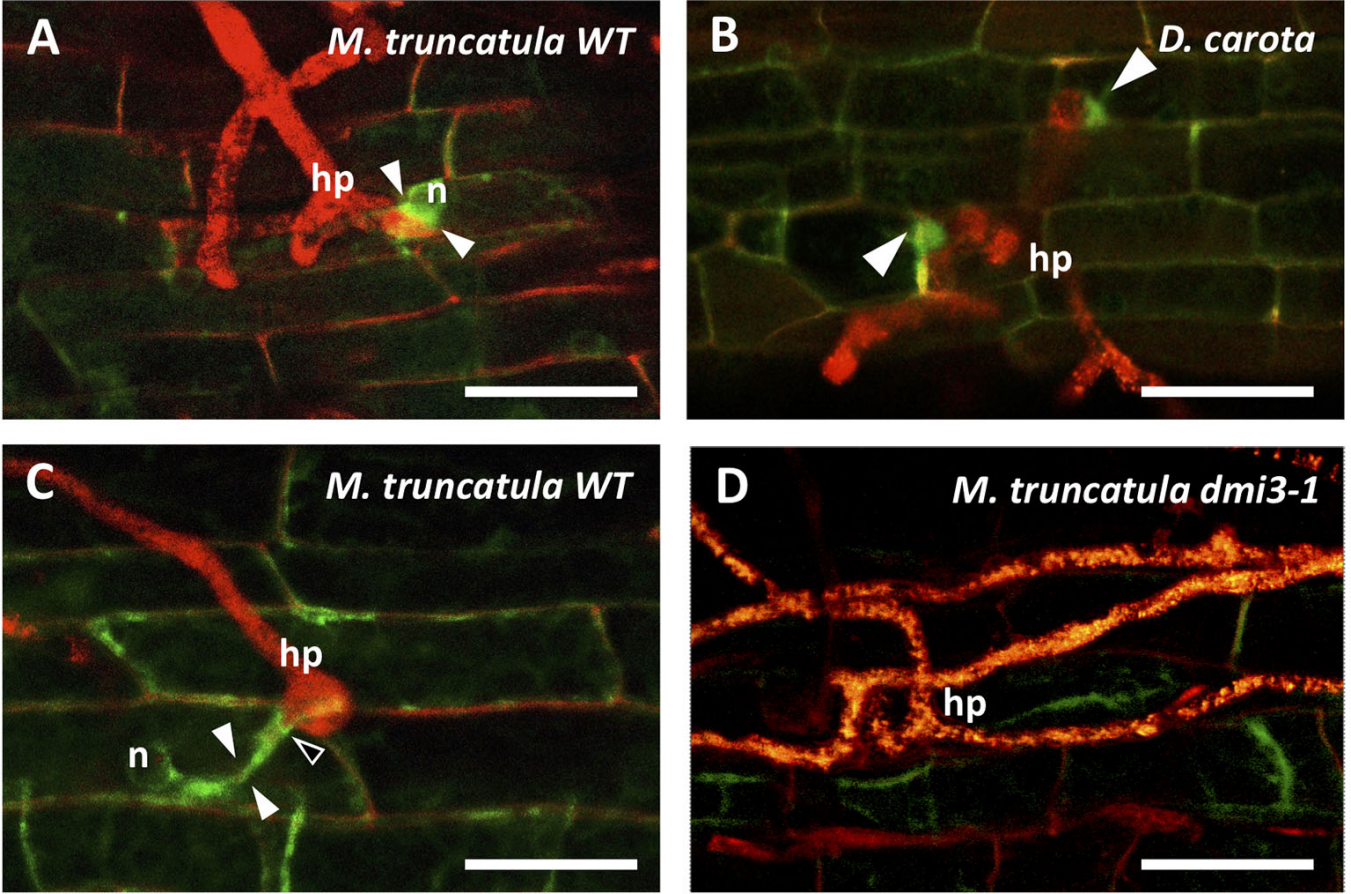
Accumulation of TPLATE-GFP in the prepenetration apparatus of *Medicago truncatula* and *Daucus carota* root epidermal cells. In the presence of a hyphopodium (hp) on the root surface of *M. truncatula* WT ROCs **(A**, **C)** and *D. carota* ROCs **(B)**, intense TPLATE-GFP fluorescence is observed in the PPA area (arrowheads), during the initial nuclear repositioning (n) **(A)**, at two sites of fungal contact within the same hyphopodium **(B)** and during the following extension of the PPA **(C)**. Furthermore, a thin line of intense fluorescence is also visible in C, outlining the point (empty arrowhead) where the penetrating hypha is developing. In contrast, no GFP accumulation is visible on the root surface at the hyphopodium contact site of *M. truncatula dmi3-1* mutants **(D)** that are impaired in PPA formation and fungal colonization. Bars = 50 µm.

A significant accumulation of TPLATE-GFP was observed in both *M. truncatula* and *D. carota* epidermal root cells in the PPA area (**Figure 1**): prior to root cell penetration the GFP signal was strong between the repositioned nucleus and the hyphopodium contact site, clearly highlighting both developing (**Figures 1A**, **B**) and fully formed PPAs (**Figure 1C**). For comparison, the fluorescence patterns of free DsRED (diffusing in the cytosol and nucleus) and PIP2-GFP (labeling a plasma membrane aquaporin) are presented in **Supplementary Figure 1**. Significantly, no intracellular accumulation of TPLATE-GFP was observed in the contacted epidermal cells of *dmi3-1* mutants (**Figure 1D**).

Due to the acknowledged role of the TPLATE complex (TPC) in endocytosis, our observations in two phylogenetically distant plants suggest that the activation of endocytic processes are part of the AM prepenetration responses in epidermal cells. This conclusion is further supported by the absence of TPLATE accumulation in *dmi3-1* mutants of *M. truncatula*, where PPAs (Genre et al., 2005) and epidermal cell penetration (Levy et al., 2004) are blocked, and *TPLATE*, *AP2A1*, and *Clathrin* are not upregulated upon AM inoculation (Russo et al., 2019).

### Perifungal Membrane Dynamics in the Cortex Recruit TPLATE

**Figure 2:**
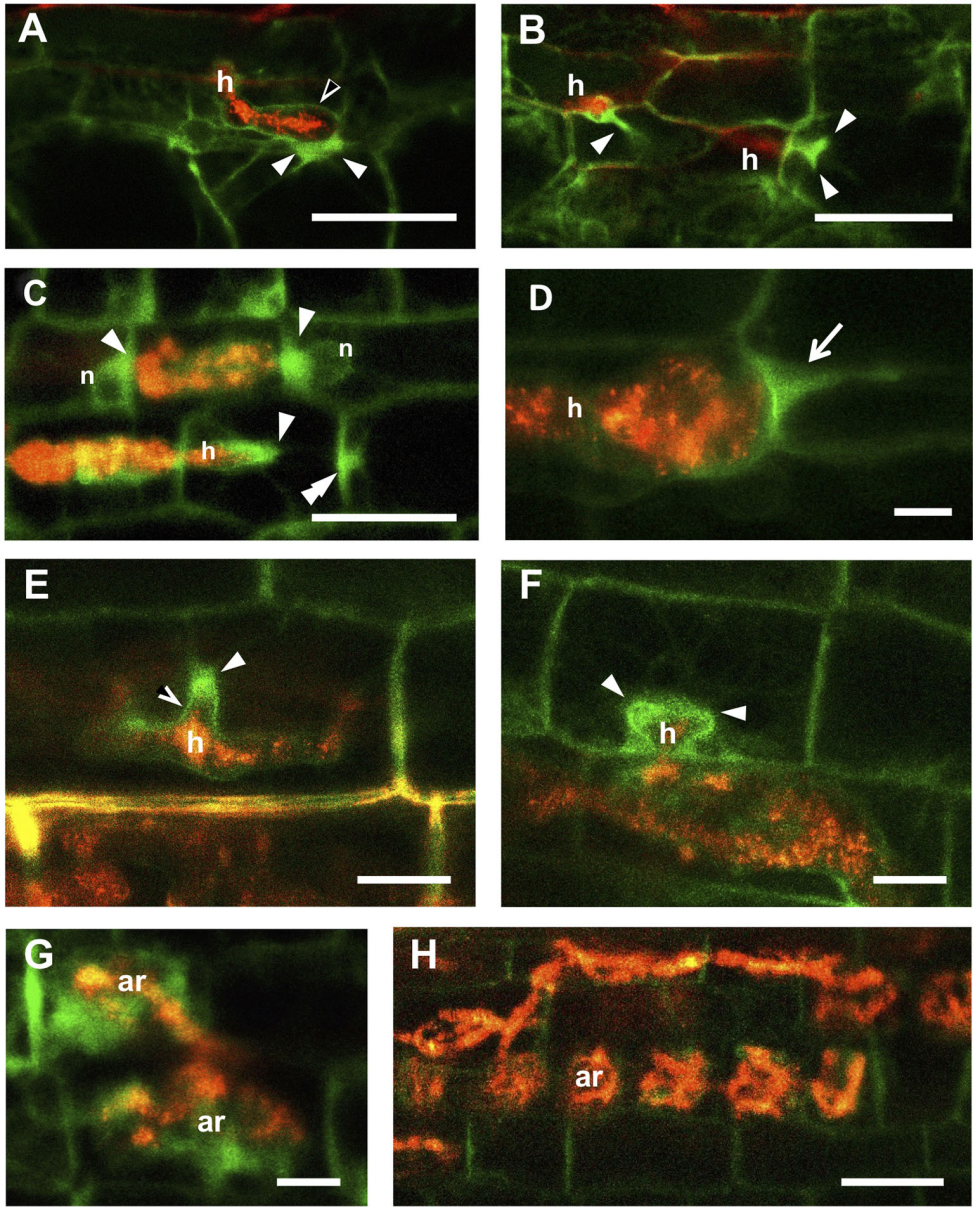
TPLATE-GFP labeling of symbiotic interface in deeper layers of *Medicago truncatula* and *Daucus carota* roots. **(A)** shows an intracellular hypha (h) inside an *M. truncatula* root epidermal cell. The perifungal membrane appears outlined by a faint GFP signal (empty arrowhead), whereas intense fluorescence accumulates in the developing PPA in the underlying outer cortical cell (arrowheads). An analogous situation is presented in **(B)**, where two hyphae (h) are imaged as they pass from cell to cell in the cortex: in both cases an intense fluorescence marks the PPAs (arrowheads). PPAs labeling at different developmental stages are also observed in the cortex of *D. carota* **(C**–**F)**. Bright GFP signals are visible around the tips and branches of intracellular hyphae (arrowheads); along the cell wall at predicted hyphal exit site (double arrowhead in **C**); and along the perifungal membrane behind the growing hyphal tip (empty arrowhead in **E**). In **(D)** a *Gigaspora gigantea* hypha is on the point of passing from one cell to the next in the same file: the GFP signal marks the typical arrow-shaped PPA (arrow) of *Paris*-type mycorrhizas. **(G)** shows two young arbuscules (ar) surrounded by diffuse GFP fluorescence, suggesting that TPLATE is also involved in arbuscule accommodation, while this intense signal is lost in cortical cells that harbor older arbuscules **(H)**. Bars = 50 µm in **(A**–**C**, **H)**; 10 µm in **(D**–**G)**.

As we extended our observations to inner root tissues, following the progress of fungal development, we remarked that intense TPLATE accumulation in PPAs was also present in outer cortical cells (**Figure 2**). In detail, by the time the penetrating hypha had reached the inner side of the epidermal cell (**Figure 2A**), an intense TPLATE-GFP labeling could be spotted in the underlying cortical cell, within the developing PPA aggregation opposite the hyphal tip. Comparably intense GFP signals could also be seen in more advanced PPAs in the outer cortex of both *M. truncatula* and *D. carota* (**Figures 2B**, **C**).

The limited translucence of *M. truncatula* ROCs (Genre et al., 2008) restricted our ability to obtain clear images of the inner cell layers. Consequently, cortex colonization was more extensively studied in the thinner and clearer *D. carota* ROCs expressing the same TPLATE-GFP fusion.

A diffuse TPLATE-GFP signal highlighted cortical PPAs of different developmental stages (**Figures 2C**, **D**), from small nucleus-associated aggregates appressed to transverse cell walls (**Figure 2C**) to broad arrow-shaped PPAs (**Figure 2D**), typical of the carrot root cortex (Genre et al., 2008), where intraradical colonization proceeds from cell to cell (*Paris*-type pattern; Dickson, 2004). Intense TPLATE accumulation was also observed around the tips of linear (**Figures 2C**, **D**) and branched hyphae (**Figure 2E**, **F**) in both outer and inner cortical cell layers. Furthermore, a fainter GFP signal extending along the perifungal membrane behind the growing hyphal tip was occasionally visible in both epidermal (**Figure 2A**) and cortical cells (**Figure 2E**).

Lastly, diffuse fluorescence was present around newly formed arbuscules that had not yet fully occupied the lumen of inner cortical cells (**Figure 2G**). By contrast, no relevant GFP accumulation was recorded around fully developed arbuscules (**Figure 2H**).

Overall, our observations of *M. truncatula* and carrot roots indicate that TPLATE-GFP is recruited to the sites of perifungal membrane development in the PPA of both plant species and strongly suggest the involvement of endocytic processes in all cell types engaged in AM colonization, before and during the development of intracellular hyphae, hyphal branches, and arbuscules.

**Figure 3:**
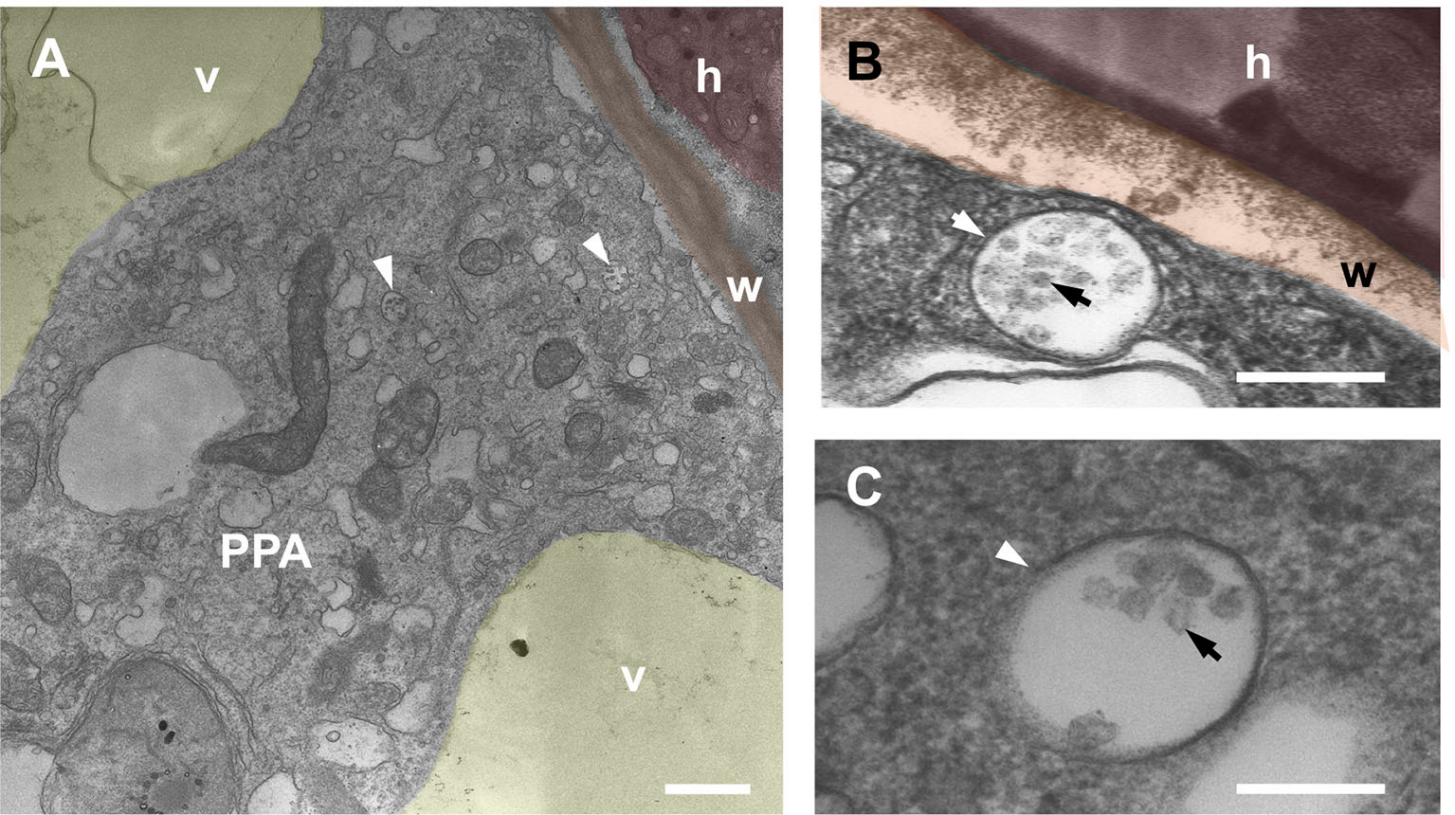
TEM imaging of multivesicular bodies within *Daucus carota* PPA aggregates. Panel **(A)** shows a PPA inside a cortical cell of *D. carota*. The cytoplasmic aggregation splitting the vacuole (v, yellow) is assembled in front of an intraradical hypha (h, red) that is contacting the cell wall (w, orange). Several multivesicular bodies (MVBs) are visible (arrows). High magnification close-ups of single MVBs are presented in panels **(B** and **C)**, highlighting the characteristic presence of an outer endosomal membrane (white arrow) and several intraluminal vesicles (black arrow). Bars = 0.8 µm in A, 0.2 µm in B, 0.1 µm in C; false colors were overlaid to the original TEM images for clarity.

This conclusion appeared in line with previous detection of MVBs in the PPA aggregate (Genre et al., 2008; Genre et al., 2012). Indeed, our new TEM observations of carrot cortical PPAs (**Figure 3**) confirmed the presence of membrane-delimited compartments of different size, containing several intraluminal vesicles, that can be ascribed with confidence to the class of MVBs, or late endosomes. Their presence appears now congruous with our localization of TPLATE-GFP in the PPA, hinting to occurrence of endocytic processes during intracellular fungal accommodation.

### TPLATE Labeling at Sites of Cell-to-Cell Hyphal Passage in Carrot

**Figure 4:**
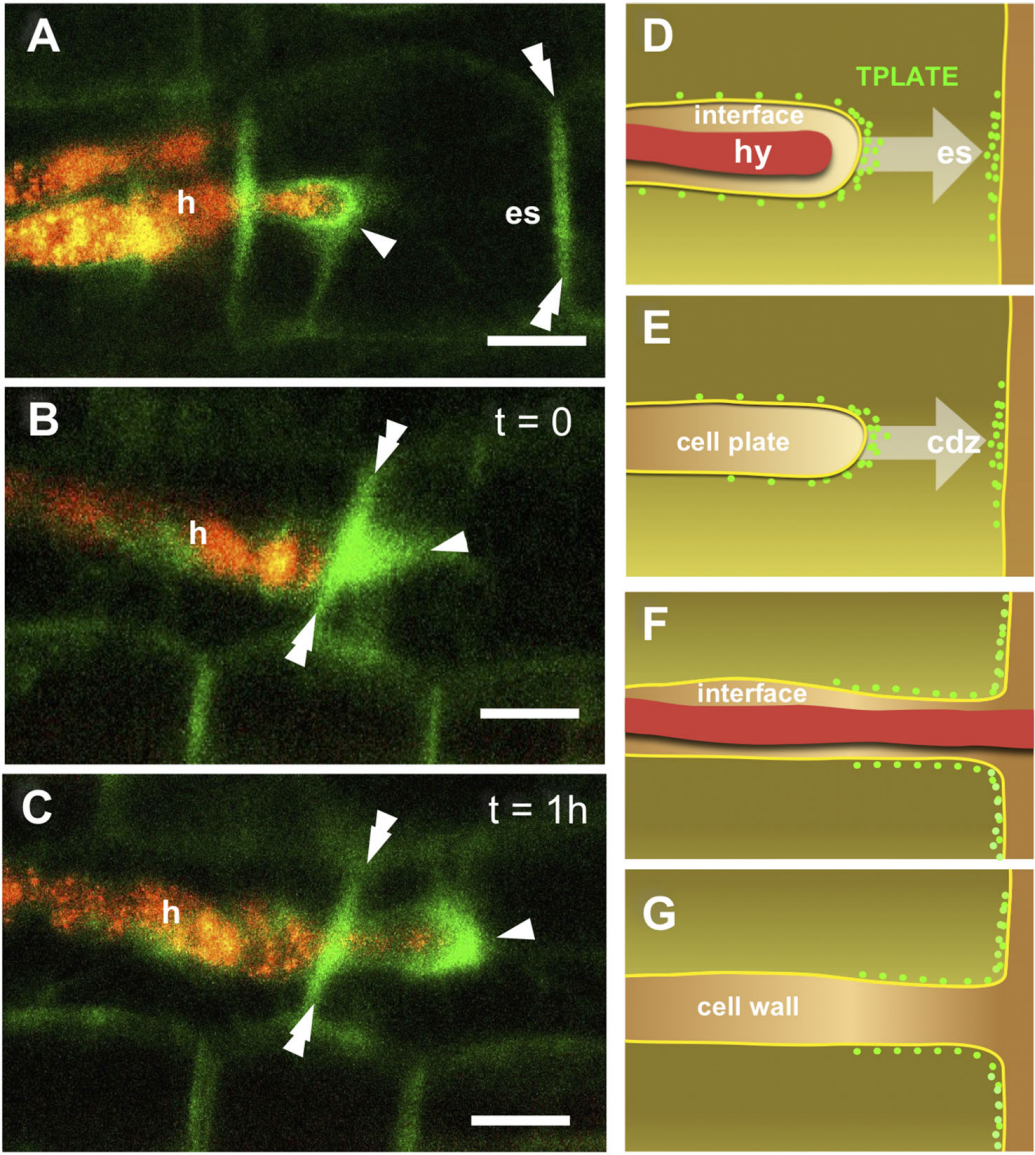
TPLATE-GFP labeling of cell-to-cell hyphal passage in the outer cortex. An intense GFP signal (arrowhead) is visible in **(A)**, around the tip of an intracellular hypha (h). This is associated with the accumulation of TPLATE-GFP along the wall (double arrowheads) that separates the colonized cell from the adjoining uncolonized cell, in the area of the predicted hyphal exit site (es). An elongated PPA (arrowhead) is visible in an uncolonized cell on the right side of panel **(B)**; the hypha growing in the cell on the left has reached the cell wall, where an intense GFP signal has accumulated (double arrowheads). The same site is pictured in **(C)** 1 h later: The hypha has now penetrated the cell on the right; a bright signal surrounds the hyphal tip (arrowheads) and a strong fluorescence is still visible along the cell wall (double arrowhead). Bars = 30 µm. Panels (**D**–**G**) show a schematic view of the analogies between perifungal membrane **(D**, **F)** and cell plate **(E**, **G)** fusion with the peripheral plasma membrane. In both cases, TPLATE accumulation was observed at the front of the developing compartment **(D**, **E)**, and at the exit site (es) or cortical division zone (cdz), respectively. Following membrane fusion **(F**, **G)**, TPLATE diffuses at the junction between the two membranes.

Tissue translucency and *Paris*-type colonization allowed a very accurate observation of fungal proliferation and associated cell responses in the outer cortex of carrot. Beside hyphal tip-associated TPLATE-GFP labeling in PPA aggregates (**Figures 4A**–**C**) a remarkable accumulation of fluorescent signal was also observed along the transverse wall corresponding to the predicted exit site (es) of the hypha from the colonized cell (**Figure 4A**). This transverse wall-associated labeling appeared to persist during and after hyphal cell-to-cell passage (**Figures 4B**, **C**). This is particularly evident when comparing the panels of **Figures 4B**, **C**, which frame the same cells as the hyphal tip grows across the transverse wall over an interval of 1 h. Such localized accumulations of membrane-associated TPLATE-GFP are clearly different from the homogeneous distribution of a membrane protein such as the aquaporin *At*PIP2 (**Supplementary Figures 1E**, **F**).

Such consistent observations hint at a role for TPC-related endocytosis in the fusion between the developing perifungal membrane and the plasmalemma at the cell exit site, a rather unusual event in plant cell dynamics that predictably involves the removal of a consistent membrane surplus.

## Discussion

### Endocytosis in Host Cell Penetration

Our previous results (Russo et al., 2019) on the upregulation of endocytic markers such as *Clathrin*, *AP2A1*, and *TPLATE* during early AM interactions, had suggested a role for clathrin-mediated endocytosis in AM fungal accommodation. Our current live-cell observations in carrot and wild-type—but not *dmi3-1*—Medicago, showing TPLATE-GFP accumulation in PPAs and along the perifungal membranes, provide more direct evidence of endocytosis in symbiotic processes of membrane remodeling and interface biogenesis for all colonized cells.

This proposed role for endocytosis in AM fungal accommodation complements previous demonstrations of the exocytic origin of the symbiotic interface (Genre et al., 2012; Ivanov et al., 2012). In fact, focused exocytic events in the plant cell are normally associated with endocytic recycling of surplus membrane (Samaj et al., 2004; Ketelaar et al., 2008). Significantly, this is the case for cell plate formation (Dhonukshe et al., 2006; Backues et al., 2007; McMichael and Bednarek, 2013) and the analogous accumulation of TPLATE at sites of perifungal membrane assembly supports the hypothesis that the whole membrane remodeling process set in motion to perform cell division is recruited by AM host cells for fungal accommodation.

Alongside membrane modeling, clathrin-mediated endocytosis is also known to be involved in receptor turnover and internalization, with the generation of signaling endosomes (Ben Khaled et al., 2015). In the context of AM interactions, it will be interesting to investigate whether this process plays a role in the perception and translocation of fungal elicitors and effectors within the host cell.

### Membrane Remodeling at Host Cell Exit Sites

While PPA-associated membrane proliferation from the site of fungal entry in the cell generates an extension of the plasmalemma (the perifungal membrane), hyphal exit from the cell lumen requires a rather different process. As the front of the proliferating perifungal membrane reaches the plasma membrane, fusion must occur between the two in order to generate a complete membrane tunnel surrounding the hypha (**Figures 4D**, **F**). Such a membrane fusion generating a trans-cellular apoplastic compartment is a peculiar event with few analogs in plant cell biology. The closest similarity is with the development of an infection thread carrying rhizobia across legume root tissues, toward a nodule primordium (Gage and Margolin, 2000; Downie, 2014). Nevertheless, 50 million year-old nitrogen-fixing symbioses are believed to have recruited part of the symbiotic responses developed earlier in the course of AM evolution, including intracellular colonization mechanisms (Bonfante and Genre, 2008). A less obvious, but very intriguing analogy is with the fusion of the cell plate membrane with the peripheral plasma membrane at the end of cell division (**Figures 4E**, **G**). In fact, the separation of daughter cells starts with the fusion of finger-like protrusions of the growing cell plate border with the plasmalemma, at the cortical division zone (Samuels et al., 1995). In the light of our current findings, the similarity between such finger-like protrusions and the tip of the perifungal membrane is striking: both membranes at the cell plate border and the cortical division zone are characterized by the active recruitment of TPC members (Van Damme et al., 2011; Gadeyne et al., 2014); our observation of TPLATE-GFP accumulation at fungal exit sites indicates the recruitment of this protein on the two fusing membranes, and is evocative of the co-optation of cell division-related membrane dynamics in symbiotic responses.

### Evolutionary-Developmental Implications

Our recent demonstration of cell cycle (Carotenuto et al., 2019a) and cell division reactivation in root cortical cells during AM colonization (Russo et al., 2019), proposed these processes as features of the 400-Myr-old AM symbiosis that were conserved for the origin of symbiotic nitrogen fixation (SNF) between legumes and rhizobia, around 50 Myr ago. While, anyway, cell cycle reactivation has been related to nodule initiation in SNF, the lack of neo-organogenesis in AM left several hypotheses open about the biological role of sparse cell divisions in the AM root cortex.

We speculated that a mechanistic analogy could link the exocytic processes of cell plate and symbiotic interface biogenesis, also based on several analogies in the formation (Lam et al., 2008; Pumplin et al., 2012) and composition in cell wall-related materials (Balestrini and Bonfante, 2014) of the two cell compartments. Our present results substantiate this hypothesis, suggesting that the cell plate deposition machinery, combining exocytic and endocytic processes, has been co-opted in symbiotic interface biogenesis at the origin of AM, and later conserved in SNF interactions.

## Data Availability Statement

The datasets generated for this study are available on request to the corresponding author.

## Author Contributions

GR designed the experiments, developed the transgenic lines, performed confocal microscopy analyses and wrote the text. GC performed transgenic line production, and contributed to the writing. VF, MaC, MiC and VV contributed to transgenic plant production and text writing. AF and PB performed electron microscopy analyses and contributed to the writing. DD provided the AtTPLATE-GFP vector and contributed to the writing. AG designed the research and experiments and wrote the text.

## Funding

Financial support for this research was granted by Compagnia di San Paolo (project REPROGRAM – Progetti di Ateneo 2012, Call 01) and UNITO grants Ricerca Locale 2013-2014-2017.

## Conflict of Interest

The authors declare that the research was conducted in the absence of any commercial or financial relationships that could be construed as a potential conflict of interest.
