# Supplementary material for: TPLATE Recruitment Reveals Endocytic Dynamics at Sites of Symbiotic Interface Assembly in Arbuscular Mycorrhizal Interactions
Source: Front Plant Sci. 2019 Dec 20;10:1628. doi: 10.3389/fpls.2019.01628 (PMC6934022; doi:10.3389/fpls.2019.01628)
Supplement: Supplementary file 1 [file Image_1.pdf]

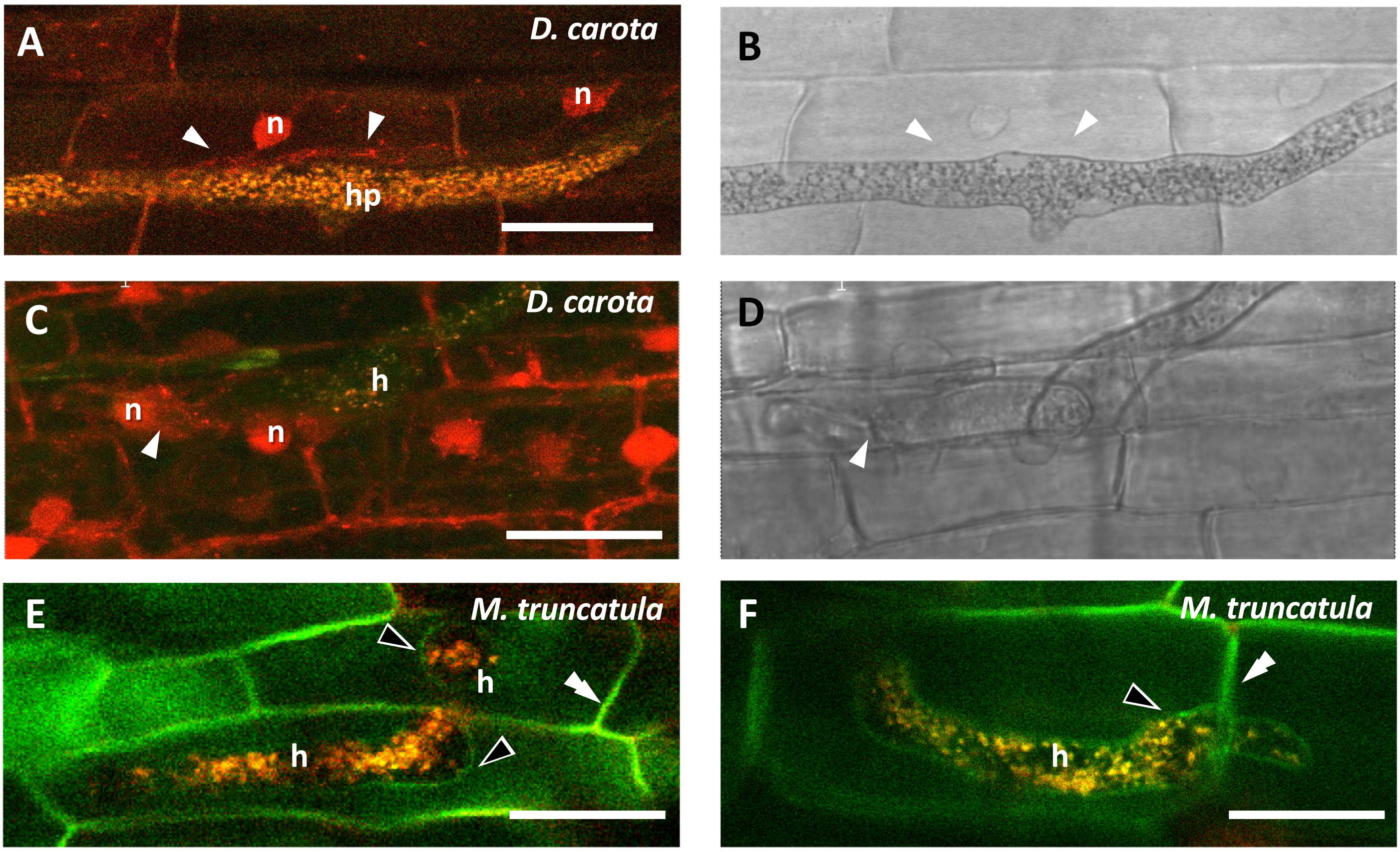

Supplementary Figure 1. Localization of fluorescent markers during root colonization elucidate the positioning of TPLATE:GFP labelling in the PPA. **A-D** Epidermal cells of *D. carota* ROCs expressing 35S::DsRED, contacted by *Gigaspora gigantea* hyphopodia: free DsRED fluorescence highlights the PPA cytoplasmic aggregation (arrowheads) both at the hyphopodium (hp) contact site (**A, B**) and after (**C, D**) hyphal penetration inside the epidermal cell. DsRED labeling of cytoplasm appears relatively uniform, with no accumulation of fluorescent signal at the perifungal membrane (**A** and **C**). **B** and **D** are transmitted light images of **A** and **C**, respectively. **E-F** Epidermal cells of *M.truncatula* ROCs expressing a 35S::*At*PIP2-GFP tag (kindly provided by D.T. Luu, Montpellier, France; Boursiac et al., 2005) are visualized at different stages of colonization with *G. gigantea*. Root cells display bright GFP signals at the plasma membrane (double arrowheads) - confirming the correct subcellular localization of the *At*PIP2 aquaporin - while the perifungal membrane around intracellular hyphae (h) show a weak, uniform labeling (empty arrowheads) with no focused accumulation. Bars 20 μm in **A-D**, 15 μm in **E** and **F**.

SUPPLEMENTARY REFERENCES

Boursiac, Y., Chen, S., Luu, D.T., Sorieul, M., van den Dries, N., Maurel, C. (2005) Early effects of salinity on water transport in *Arabidopsis* roots. Molecular and cellular features of aquaporin expression. *Plant Physiol* 139, 790–805. doi: 10.1104/pp.105.065029
